# Supplementary material for: Below Average Cognitive Ability—An under Researched Risk Factor for Emotional-Behavioural Difficulties in Childhood
Source: Int J Environ Res Public Health. 2021 Dec 8;18(24):12923. doi: 10.3390/ijerph182412923 (PMC8702024; doi:10.3390/ijerph182412923)
Supplement: Supplementary file 1 [file ijerph-18-12923-s001.zip › ijerph-1469280-supplementary.pdf]

Supplementary Material

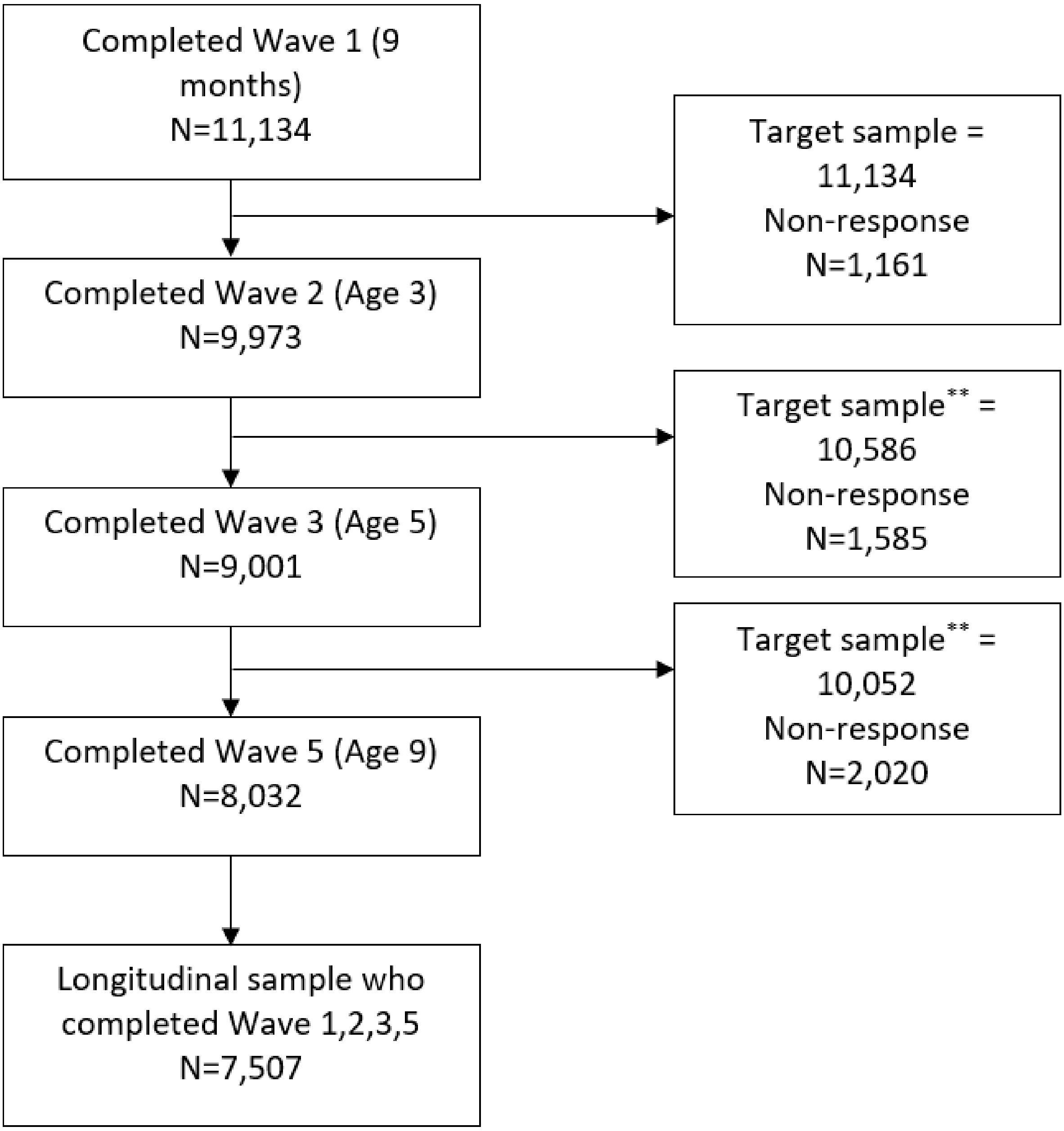

Figure S1. Participation in Growing Up in Ireland Study

* Wave 4 of the study consisted of a postal questionnaire only, which 5,344 participants completed.

** Target samples included those who participated in the previous round and those who had participated at one of the earlier rounds of the study

Figure S1 Legend

Flow diagram showing participants at each Wave of the Growing Up in Ireland Survey. A total of 11,134 participants were recruited at Wave 1. At Wave 2, the target sample was the same 11,134 participants. At Wave 3 and Wave 5 the target sample included those who had completed the previous wave as well as those who had completed one of the earlier rounds. There were 7,507 participants who completed Wave 1,2,3 and 5 of the study. Wave 4 of the study consisted of a postal

questionnaire only, which 5,344 participants completed. Data from Wave 4 was not included in this study. A weighting variable calculated by the Growing Up in Ireland Team was applied to this population of 7,507 to ensure the structure was aligned with that of the population across key sociodemographic variables.

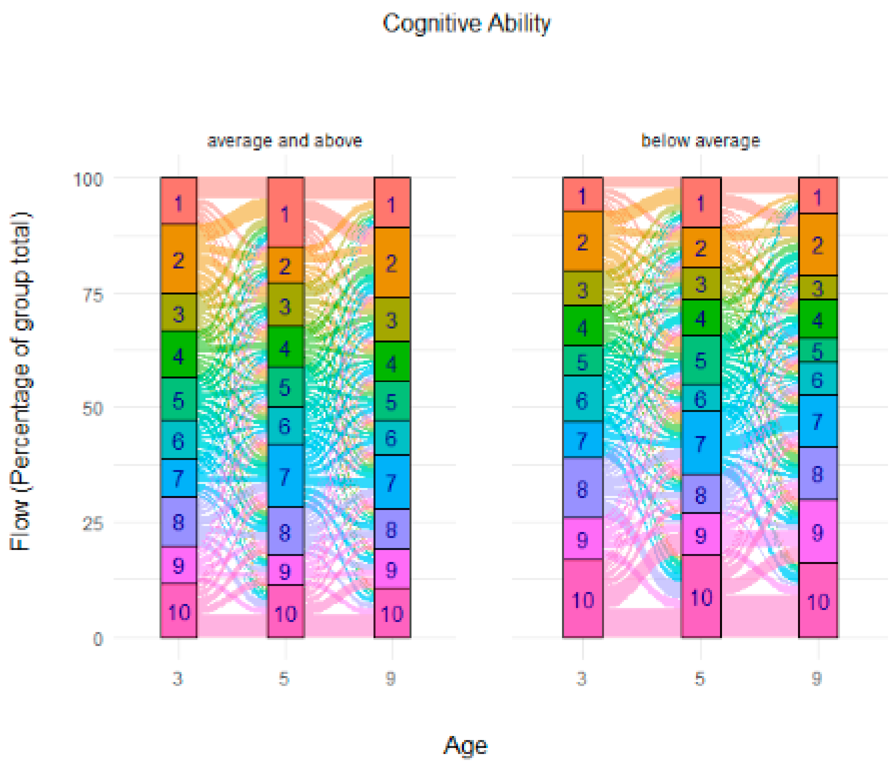

Figure S2. Alluvial Plots of SDQ Decile Scores at age 3, 5, and 9 Years According To Cognitive Ability.

Figure S2 Legend
Alluvial Plots of SDQ Decile Scores at Age 3, 5, and 9 Years According to Cognitive Ability. Total SDQ Scores were split into deciles with decile 10 representing the highest 10% of total SDQ scores at that time point and decile 1 representing the lowest 10% of total SDQ scores at that time point. Each flow represents the number of participants in that flow, with the width of the flow proportional to the number of participants. At age 3, 5, and 9 years a larger proportion of those in the below average cognitive ability group had SDQ scores in the highest two deciles.

Table S1. General Linear Model Examining Cognitive ability as a predictor of Change in Total SDQ Score

	Model 5<br>Interaction effect<br>Cognitive ability x Age	Model 6<br>Adjusted for child factors x Age	Model 7<br>Adjusted for child factors and maternal depression x Age	Model 8<br>Adjusted for child factors, maternal depression, and sociodemographic factors x Age
Intercept	7.32 (7.21 – 7.42)			
Below average cognitive ability: Age 5	0.05 (-0.29 – 0.39)	0.02 (-0.32 – 0.36)	0.01 (-0.33 – 0.35)	0.01 (-0.33 – 0.35)
Below average cognitive ability: Age 9	0.60 (0.19 – 1.00)	0.52 (0.11 – 0.93)	0.50 (0.10 – 0.91)	0.47 (0.06 – 0.87)
Participants	7,079	7,079	7,079	7,079
Observations	21,237	21,237	21,237	21,237
Marginal/ Conditional R2	0.007 / 0.926	0.016 / 0.926	0.051 / 0.928	0.079 / 0.933
BIC	120230.1	120182.6	119841.0	119616.9
p-value		<0.001	<0.001	<0.001

Model 6 adjusted for interaction terms including child gender and gestational age

Model 7 adjusted for interaction terms including child gender, gestational age, maternal depression

Model 8 adjusted for interaction terms including child gender, gestational age, maternal depression, presence of a partner, maternal education
